# Self-Supervised Three-Dimensional Ocean Bottom Node Seismic Data Shear Wave Leakage Suppression Based on a Dual Encoder Network

**DOI:** 10.3390/s25030682

**Published:** 2025-01-23

**Authors:** Zhaolin Zhu, Zhihao Chen, Bangyu Wu, Lin Chen

**Affiliations:** 1 Hainan Institute, Zhejiang University, Sanya 572025, China; 22234017@zju.edu.cn; 2 School of Mathematics and Statistics, Xi’an Jiaotong University, Xi’an 710049, China; bangyuwu@xjtu.edu.cn (B.W.); 3122307006@stu.xjtu.edu.cn (L.C.)

**Keywords:** OBN, deep learning, U-Net, shear wave, noise suppression

## Abstract

Ocean Bottom Node (OBN) is a seismic data acquisition technique, comprising a hydrophone and a three-component geophone. In practice, the vertical component is susceptible to high-amplitude, low-velocity, and low-frequency shear wave noise, which negatively impacts the subsequent processing of dual-sensor data. The most commonly used method is adaptive matching subtraction, which estimates shear wave noise in the vertical component by solving an optimization problem. Neural networks, as robust nonlinear fitting tools, offer superior performance in resolving nonlinear mapping relationship and exhibit computational efficiency. In this paper, we introduce a self-supervised shear wave suppression approach for 3D OBN seismic data, using a neural network in place of the traditional adaptive matching subtraction operator. This method adopts the horizontal components as the input to the neural network, and measures the output and the noisy vertical component to establish a loss function for the network training. The network output is the predicted shear wave noise. To better balance signal leakage and noise suppression, the method incorporates a local normalized cross-correlation regularization term in the loss function. As a self-supervised method, it does not need clean data to serve as labels, thereby negating the tedious work of obtaining clean field data. Extensive experiments on both synthetic and field data demonstrate the effectiveness of the proposed method on shear wave noise suppression for 3D OBN seismic data.

## 1. Introduction

The continuous exploitation and utilization of offshore oil and gas resources has facilitated the rapid advances in offshore exploration technology. Exploration and development targets are gradually moving towards deep, complex tectonic and lithological oil and gas reservoirs, necessitating high-quality seismic data [1,2]. Ocean bottom node (OBN) is a marine multi-component acquisition technology, which was developed after ocean bottom seismometers and ocean bottom cables (OBCs) and facilitates the exploration of tectonically complex areas. The sources of OBN have a dense grid distribution and the receivers are relatively sparsely distributed. Therefore, OBN offers distinct advantages over marine towed-streamer acquisition technology, notably its flexibility in deployment and ability to consistently produce high-fidelity seismic data [3,4]. In addition, the wide offset and azimuth geometry of the OBN are critical to image the underlying strata shielded by high-impedance geological bodies, such as salt domes [5]. Consequently, OBNs are increasingly applied in areas such as oil and gas exploration, seismic monitoring, and deep structure detection [6]. However, the processing of OBN seismic data has its own challenges, one of which is the suppression of shear wave leakage in the vertical component of the geophone.

OBNs consist of a hydrophone, which records the pressure wavefield, and three geophones (one vertical and two horizontal) for elastic wavefield recording [7,8]. Ideally, the vertical component of geophone receives P-waves and the horizontal component receives S-waves [9]. However, the vertical component often suffers shear wave leakage from the horizontal components. In common receiver gathers (CRGs), the shear wave shows a coherent characteristic, while it presents an incoherent characteristic in common shot gathers [10]. Experts have studied the causes of shear wave leakage, and the most plausible explanation seems to be the presence of strong wavefield scattering in shallow sediments [11,12]. This seriously affects the subsequent processing of OBN data, including the matching of the vertical component of the geophone with the hydrophone component (i.e., PZ summation). PZ summation could feasibly produce a pure up-going wave, subsequently suppressing ghost waves, which is one of the advantages of OBN acquisition [13,14,15].

For noise attenuation in seismic data, traditional denoising methods include transform domain filtering [16,17,18,19], decomposition [20,21,22], and prediction filtering [23,24,25], etc. Some of those methods have been used for shear wave suppression. Shatilo et al. [26] achieved S-wave suppression in the *f*-*k* domain, subsequently applying normal-moveout correction (NMO) to streamline the filter design. Craft and Paffenholz [27] matched the envelopes of the geophone data and hydrophone data for better noise suppression after the 3D τ-p transform. Yu et al. [28] used the local properties in the 2D wavelet domain to simultaneously achieve amplitude matching and noise removal. Following [28], Peng et al. [29] employed an enhanced 2D wavelet transform for shear wave attenuation, thereby improving the denoising effect. Pool et al. [30], Jiang et al. [31], and Perrone et al. [32] achieved the wavefield separation of multi-component data in the Radon domain. Ren et al. [33] used P/Z amplitude ratio thresholding to mitigate shear wave noise in the dual-tree complex wavelet transform domain. Yang et al. [34] and Ren et al. [35] decomposed the data into different frequency bands and dipping angles in the curvelet domain to achieve simultaneous PZ matching and shear wave suppression. Later, the aforementioned method was also successfully applied to 3D data processing [35]. A singular value decomposition (SVD)-based shear wave noise attenuation method driving using hydrophone data was proposed by Roodaki et al. [36]. Hampson et al. [37] employed a distributed compressive sensing technique to simultaneously separate the up–down wavefield and the shear wave noise suppression. Adaptive matching filtering is also a common way to deal with shear wave leakage. Wang [38] used the horizontal component signal as reference noise to adaptively suppress shear wave interference. Similarly, Jeong et al. [39,40] employed a mask operator to protect the vertical component signal from signal leakage before adaptive matching, and finally designed calibration filters to recover the P and Z amplitudes. The above mentioned techniques have been effectively applied to suppress field OBN data shear wave leakage.

Recently, artificial intelligence technology, especially deep neural networks (DNNs), has made remarkable achievements in seismic processing and interpretation. The application scenarios include but not limited to random noise suppression [41,42,43,44], coherent noise suppression [45,46,47], fault detection [48], seismic inversion [49], salt segmentation [50], and earthquake detection [51,52,53]. Deep learning methods are divided into three categories: supervised, unsupervised, and self-supervised. In addressing shear wave leakage suppression, there have been several notably successful applications using supervised deep learning methods. Sun et al. [12] used the hydrophone P-component as the noise-free dataset and the radial (R) horizontal component as the representative of shear wave noise to train the DNN. A trained network was then applied to the vertical geophone component for shear wave suppression. In addition similar denoising results were achieved on U-Net and ResNet architectures. Wang et al. [54] introduced an innovative strategy for generating training sets. During the training process, it dynamically adjusts the scaling factor, controlling the amplitude of the horizontal component in the network input based on the denoising results. Although the method of [12,54] does not require clean vertical component data, its training is time-consuming and it faces issues regarding generalization to 3D data. On the other hand, unsupervised and self-supervised methods eliminate the need for ground truth to serve as labels, thus avoiding the issue of labels being lacking in the field data. Furthermore, they remove the necessity of a large amount of clean–noisy pairs in network training [55]. Chen et al. [56] proposed a self-supervised method for 2D OBN shear wave suppression. This method takes the horizontal components’ data as inputs; then, the output of the network is used to calculate the loss with the noisy vertical component data and continuously optimize the network. Ultimately, the network outputs the shear wave noise in the vertical component.

In this paper, following [56], we propose a self-supervised deep learning method for shear wave suppression in 3D OBN seismic data. The efficiency is enhanced by the dual encoder, which can process noise from two horizontal components simultaneously. This method transforms the solution of the adaptive filter coefficients to optimize the neural network parameters. Subsequently, the network parameters are optimized by calculating the loss function similarly to the least squares problem. Finally, the denoised vertical component signal is obtained by subtracting the estimated shear wave noise (i.e., the output of the network). Considering the three-dimensional data to be processed, a lightweight U-shaped network with three downsample steps is selected. In order to achieve a better separation of signal and noise, a convolutional block attention module (CBAM) [57] is introduced into the U-shaped network, which enables the network to better extract the noise details. In addition, a local normalized cross-correlation (LNCC) regularization term is incorporated to the basic MSE loss function [58]. This is effective for multiple wave separation, which was validated in the traditional methods [50,59,60,61]. The regularization allows for a better balance between signal leakage and noise suppression. In experiments on 3D synthetic and field data, the proposed method is compared with a method based on U-Net, demonstrating its superiority and effectiveness. The main contributions of this paper are as follows:

1. For shear wave suppression in 3D OBN seismic data, this paper proposes a self-supervised method requiring only three-component geophone data. This method designs a U-shaped network with a dual encoder structure so that the two horizontal components are input through two encoders. This suppresses the two sources of horizontal leaked noise simultaneously to improve the computational efficiency.
2. To enhance the extraction of low-dimensional features, the proposed method integrates an attention mechanism into the skip connection. This contains channel attention and spatial attention to better acquire features from both dimensions.
3. To balance signal leakage and noise suppression, the local normalized cross-correlation regularization is incorporated into the basic MSE loss. This hybrid loss function not only ensures network stability but also mitigates the risk of over-fitting.

## 2. Methodology

In this section, an overview of the shear wave leakage problem is presented, followed by the details of the proposed method for shear wave suppression. The proposed method integrates CBAM into the skip connection to better capture the features of the seismic data. Finally, a regularization term related to wave field features is introduced to achieve better denoising results.

### 2.1. Shear Wave Leakage Problem Formulation

**Figure 1:**
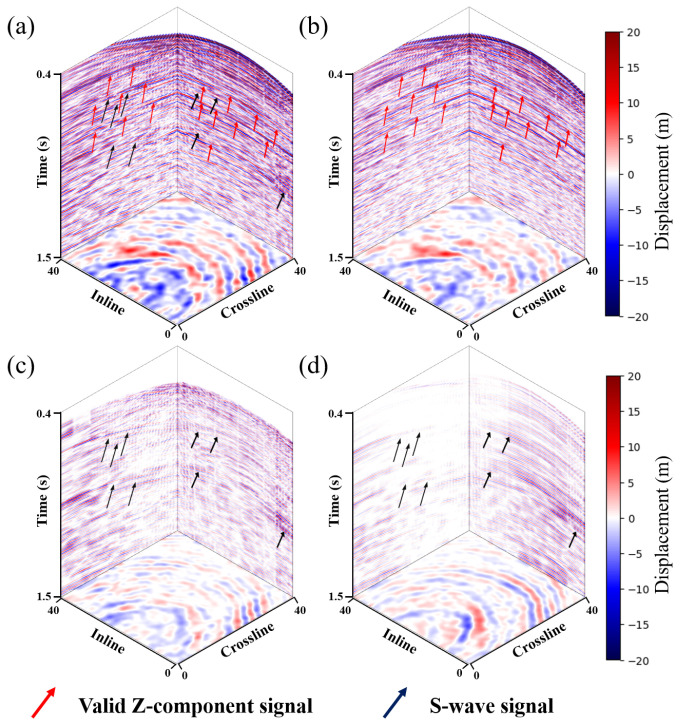
Field data demonstration of the shear wave suppression problem. (**a**) Noisy vertical component. (**b**) Denoised vertical component. (**c**) Predicted shear wave noise. (**d**) Horizontal component.

Owing to the poor coupling of the geophone to the seafloor, as well as other factors, the vertical component of the geophone may contain shear wave leakage. Assume Z ∈ $R^{T\times H\times W}$ is the noisy vertical Z-component data, where *T* denotes the number of time samples, and *H* and *W* denote the trace indexes in the two space dimensions. The corresponding mathematical expression for Z is as follows:

(1) $$Z=Z_{p}+X_{l}+Y_{l},$$

where $X_{l}$ and $Y_{l}$ are the horizontal X-component and Y-component shear wave leakage. The shear wave leakage suppression is to estimate the shear wave noise in the Z-component and subtract it to obtain the clean Z-component data $Z_{p}$. An example of shear wave leakage suppression is presented in Figure 1. It can be seen clearly (Figure 1a) that the shear wave noise in the vertical component has a larger dip angle compared to the valid Z-component event. The morphology of the shear wave noise (Figure 1c) is same as that of the horizontal component signals (Figure 1d).

### 2.2. Review of Adaptive Matching Subtraction

For the shear wave suppression, the industry usually uses methods based on adaptive matching subtraction [62], which can be expressed as follows:

(2) $$Z_{p}=Z-\alpha_{X}⊙X-\alpha_{Y}⊙Y,$$

where X and Y are the horizontal X- and Y-components. This method can handle complex field seismic data with temporal and spatial variations more effectively and avoid overlap between signal and noise in the high-dimensional domain. The coefficients $\alpha_{X}$, $\alpha_{Y}$ of the filter are typically obtained by solving the following optimization problem:

(3) $$\begin{array}{c} {\hat{\alpha }}_{X},{\hat{\alpha }}_{Y}=\arg\min_{\alpha_{X},\alpha_{Y}}{∥Z-\alpha_{X}⊙X-\alpha_{Y}⊙Y∥}_{2}^{2}\\ \;+R(\alpha_{X},\alpha_{Y}), \end{array}$$

where ${\hat{\alpha }}_{X}$ and ${\hat{\alpha }}_{Y}$ are the estimated matching filtering coefficients. *R* denotes a regularization operator, which can better control the stability of the solution. Usually, the regularization terms include Tikhonov regularization [63] and shaping regularization [64]. Equation (3) can be solved using the conjugate gradient method.

### 2.3. Deep Learning Method

The deep learning method establishes a nonlinear mapping relationship through specific network structures and optimizes network parameters with appropriate loss functions. Inspired by [56], this method uses DNN to realize adaptive matching subtraction with an appropriate loss function. The loss function can be expressed as follows:

(4) $$L\left(\theta_{X},\theta_{Y}\right)={∥Z-F_{X}\left(X,\theta_{X}\right)-F_{Y}\left(Y,\theta_{Y}\right)∥}_{2}^{2},$$

where $F_{X}$ and $F_{Y}$ represent the networks corresponding to the X and Y components, respectively. $\theta_{X}$ and $\theta_{Y}$ are the parameters of two networks. In our approach, a dual-encoder structure is chosen for efficient processing so that the two horizontal components can be input simultaneously. Therefore, the loss function can be further expressed as follows:

(5) $$L\left(\theta \right)={∥Z-F\left(\left(X,Y\right),\theta \right)∥}_{2}^{2},$$

where F denotes a U-shaped network with a dual encoder. θ represents the network parameters. The next section provides details of the deep learning method.

### 2.4. Network Structure

**Figure 2:**
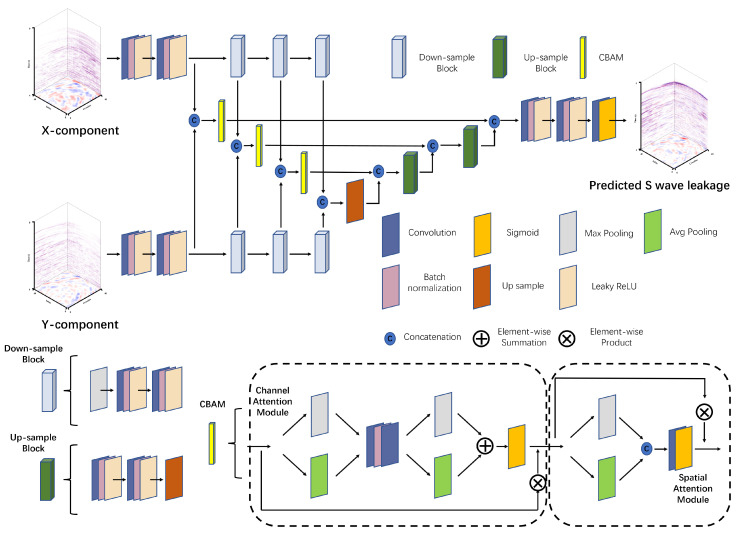
A dual-encoder -based network structure was used for the shear wave suppression problem, which consists of a downsample block, upsample block, and convolution block attention module (CBAM). The CBAM contains CAM and SAM. The inputs to the network were two horizontal components, i.e., the X- and Y-components. The output was the predicted noise.

A suitable network structure is the key to deep learning methods. The proposed method selects the U-shaped network as the basic network [65], as shown in Figure 2. This has two parts, an encoder and a decoder. To improve the processing efficiency, this method replaces the single-encoder structure of the U-Net with two encoders to process the two horizontal components simultaneously. The network inputs include two parts: the X and Y horizontal component. Then, the loss function is calculated using the noisy vertical component and the network output to optimize the network parameters, as shown in Equation (5). Thus, the mapping relationship of the network is expressed as follows:

(6) S=F((X,Y),θ),

where S is the output of the network, i.e., the shear wave noise estimated by the network.

The encoder, i.e., the left half of the network, uses two branches to extract features from two horizontal components. Every branch has the same structure, which consists of multiple down-sample modules. These are used for feature extraction to obtain better contextual information. Each down-sample module contains two 3D convolutional layers and one 3D max-pooling layer with a 2 × 2 × 2 pooling size. The convolutional layer contains a convolutional operator with a convolutional kernel of 3 × 3 × 3 and 1 × 1 × 1 stride size, a batch normalization (BN) layer, and an activation function layer. The BN layer accelerates the convergence of the network, facilitating the training process and reducing the initialization demands on the network. The activation function plays a critical role in ensuring that the network achieves a nonlinear mapping. Thus, the output of the convolutional layer can be defined as follows:

(7) $$\tilde{X}=\sigma \left(BN\left(X*W+b\right)\right),$$

where ∗ denotes the convolution operation, W and *b* are the weight matrix and bias vector, respectively. In our method, the activation function σ is LeakyReLU, with the following expression:

(8) $$\begin{array}{c} \mathrm{LeakyReLU}\left(x\right)=\left\{\begin{array}{c} x,\;\mathrm{if}\;x\geq 0\\ \beta x,\;\mathrm{if}\;x<0 \end{array}\right. \end{array}$$

where 0<β≤1. In our method, β is chosen as 0.1. Compared to ReLU, our method can implement back-propagation for negative input values. The right half of the network is the decoder, which consists of multiple upsample modules and is used to recover the data. Each upsample module contains two convolutional layers and one upsample layer. In the network, the upsample operation is trilinear interpolation. The final layer of the network restores the features to the predicted shear wave noise through a 3 × 3 × 3 convolution layer. Before the convolution layer, the proposed method maps the output values to [0,1] using the Sigmoid activation function.

The attention mechanism is an architecture that emulates the human visual and cognitive systems, enabling neural networks to focus on relevant features during processing. By introducing the attention mechanism, neural networks can automatically learn and selectively focus on important information, improving the performance and generalization ability of the model. Our method introduces a convolutional block attention module (CBAM) that combines the channel attention module (CAM) and spatial attention module (SAM), as shown in Figure 2; this can effectively improve the performance of the network without requiring a large number of parameters. In the first stage of CBAM, the CAM puts the input tensors through global max pooling and global average pooling, respectively. Then, their outputs are fed into a two-layer neural network (MLP) with shared network parameters. After that, the features that are output from the MLP are subjected to element-wise summation, followed by a Sigmoid activation to generate the final channel attention feature. Then, the input and output of the channel attention are subjected to an element-wise product to obtain the output of the first part. The output of the previous step is used as the input to the SAM. In the second stage (i.e., SAM), the input features are first processed through channel-based global max pooling and global average pooling and are concated in the channel dimension. Then, a 7 × 7 × 7 convolution operation and Sigmoid activation function is performed to generate the spatial attention feature, and finally the output features and the input of the module obtain the output of the final CBAM module thruogh the element-wise product. Regarding the order of CAM and SAM, Ref. [57] demonstrated through extensive experiments that placing CAM before SAM yields the best results.

### 2.5. Loss Function

As mentioned above, this paper chooses the L2 norm as the main component of loss function. In order to better protect the effective signal and improve the stability of the network, the local normalized cross-correlation (LNCC) regularization is introduced into the loss function with the following expression:

(9) $$\begin{array}{cc} \mathrm{LNCC}(A,B) & =\sum_{i=1}^{n}{\left(\frac{\mathrm{Cov}(A_{i},B_{i})}{\sqrt{\mathrm{Var}\left(A_{i}\right)\mathrm{Var}\left(B_{i}\right)}}\right)}^{2}, \end{array}$$

where Cov and Var denote covariance and variance, respectively. In our problem, *A* and *B* correspond to the predicted noise and the denoised signal, respectively. $A_{i}$ and $B_{i}$ represent the local window data of *A* and *B* with a size of 9 × 9.

The proposed method uses LNCC to calculate the cross-correlation between the predicted noise and the denoised signal, thereby enhancing the denoising performance of the network. A higher cross-correlation indicates incomplete noise suppression, while a lower cross-correlation means a better separation of noise and signal. Consequently, the loss function is expressed as follows:

(10) $$\begin{array}{cc} L\left(\theta \right) & ={∥Z-F((X,Y),\theta )∥}_{2}^{2}+\lambda L_{LNCC}\left(Z-F((X,Y),\theta ),F((X,Y),\theta )\right), \end{array}$$

where λ is the weighting factor.

In addition, before training the network, data are normalized to [0,1] using the min–max normalization method, the expression is as follows:

(11) $$\begin{array}{c} \tilde{d}=\frac{d-d_{min}}{d_{max}-d_{min}}, \end{array}$$

where *d* and $\tilde{d}$ represent the normalized and de-normalized data, respectively. $d_{max}$ and $d_{min}$ are the maximum and minimum values in the three-component data. Our method does not divide the data into smaller patches. Since the shear wave leakage is not globally relevant, the 3D data volume is used as an input to the network.

## 3. Experiment

In this section, the effectiveness of the proposed method is verified through the synthetic and field data, which can effectively suppress the shear wave noise in the 3D OBN seismic data and demonstrate that the method can be used as an alternative to adaptive matching subtraction. Experiments with all deep learning methods were conducted on an NVIDIA A100 Tensor Core GPU with 80 GB memory.

### 3.1. Evaluation Metrics

To better evaluate the performance of the proposed method, this paper chooses three evaluation metrics that are commonly used in the scenario of seismic denoising, such as structural similarity (SSIM) [66], signal-to-noise ratio (S/N), and local similarity (LS) [67]. This allows for a quantitative comparison of the performance of each method according to the corresponding metrics.

Firstly, the SSIM can be expressed as follows:

(12) $$\mathrm{SSIM}\left(x,y\right)=\frac{\left(2\mu_{x}\mu_{y}+C_{1}\right)\left(2\sigma_{xy}+C_{2}\right)}{\left(\mu_{x}^{2}\mu_{y}^{2}+C_{1}\right)\left(\sigma_{x}^{2}+\sigma_{y}^{2}+C_{2}\right)},$$

where $\mu_{x}$ and $\mu_{y}$ represent the means of *x* and *y*, respectively; $\sigma_{x}$ and $\sigma_{y}$ denote the variances of *x* and *y*, respectively; $\sigma_{xy}$ is the covariance of *x* and *y*; and $C_{1}$ and $C_{2}$ are two constants. The closer the SSIM value to 1, the better the denoising performance.

And the S/N can be expressed as follows:

(13) $$S/N=10\log_{10}\left(\frac{{∥S∥}^{2}}{{∥\bar{S}-S∥}^{2}}\right),$$

where $\bar{S}$ represents 3D noisy or denoised data and *S* represents 3D clean data. A higher S/N indicates a better denoising effect.

LS is used as the evaluation metric in the field data experiment, which denotes the noise suppression effect by calculating the similarity between the horizontal components and the predicted noise. A higher local similarity demonstrates better noise suppression.

### 3.2. Synthetic Data Example

**Figure 3:**
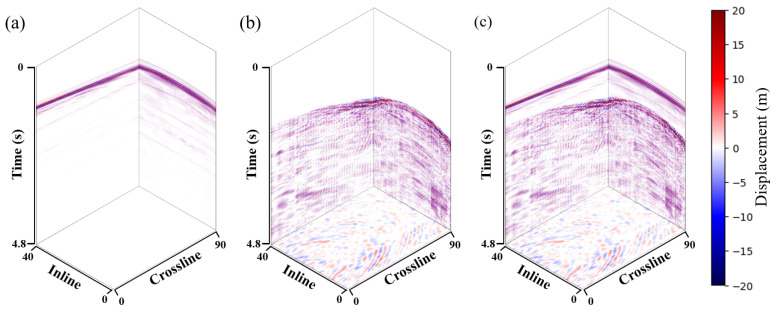
3D synthetic data example. (**a**) Clean P-wave signal. (**b**) Shear wave noise predicted by traditional method. (**c**) Noisy P-wave signal.

This paper first demonstrates the validity of the proposed method through synthetic data. Because the vertical component of the geophone mainly receives the P-wave signal in the field data, our method chooses the *SEG C3 45 shot* (https://wiki.seg.org/wiki/SEG_C3_45_shot accessed on 20 July 2024) dataset as the clean P-wave signal in the synthetic data. Then, the shear wave noise predicted by commercial software is added to the clean P-wave signal to obtain noisy data, as shown in Figure 3. According to the proposed method, the two horizontal components are inputted into the network as a noise model to predict the shear wave leakage. The following hyperparameters were set in the synthetic experiment. The initial learning rate was set to 0.001, and then, after 500 epochs, the learning rate was reduced to half of its original value. The weighting parameter λ was set to 0.001. Our network was trained for a total of 800 epochs.

**Figure 4:**
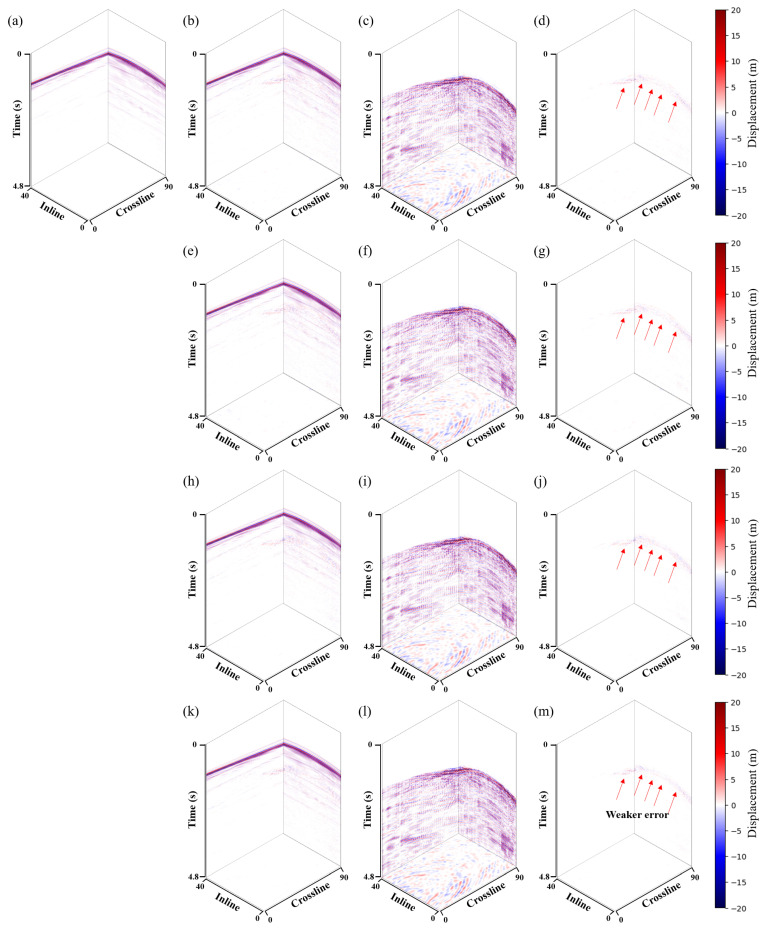
3D synthetic data shear wave suppression results. (**a**) Clean data. Denoised data using the (**b**) *Unet*, (**e**) *Unet-LNCC*, (**h**) *Unet-CBAM* and (**k**) proposed method. Removed shear wave noise using the (**c**) *Unet*, (**f**) *Unet-LNCC*, (**i**) *Unet-CBAM* and (**l**) proposed method. (**d**,**g**,**j**,**m**) correspond to the error of the denoised and clean data.

The proposed method used a U-shaped network with three downsamples as a baseline. LNCC regularization and the CBAM module were added separately to verify the effectiveness of the proposed method. This paper named these methods *Unet*, *Unet-LNCC*, and *Unet-CBAM*. Figure 4 shows the denoising effect of the four methods on the 3D synthetic data. Overall, all methods effectively suppress shear wave noise. However, in areas where the noise distribution is strong and closely orientated with the signal, the proposed method exhibits a better denoising performance with less residual noise, as indicated by the red arrows in Figure 4m. Figure 4d,g,j and m represent the differences between the denoised data and the clean data. It can be seen that the proposed method contains fewer errors.

**Figure 5:**
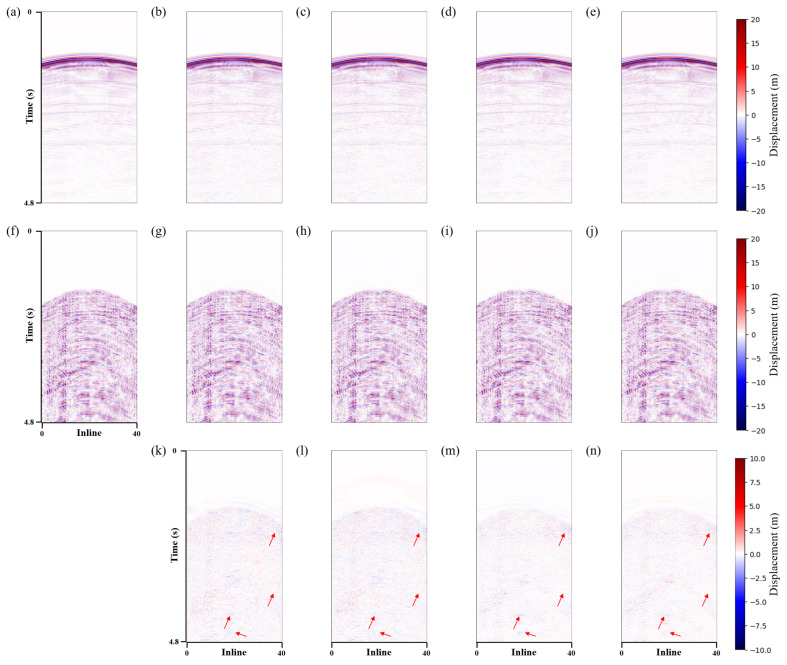
Synthetic data crossline profile shear wave suppression results. (**a**) Clean data. Denoised data using the (**b**) *Unet*, (**c**) *Unet-LNCC*, (**d**) *Unet-CBAM* and (**e**) proposed method. (**f**) Field shear wave noise. Removed shear wave noise using the (**g**) *Unet*, (**h**) *Unet-LNCC*, (**i**) *Unet-CBAM* and (**j**) proposed method. (**k**–**n**) correspond to the difference of the denoised and clean data.

**Figure 6:**
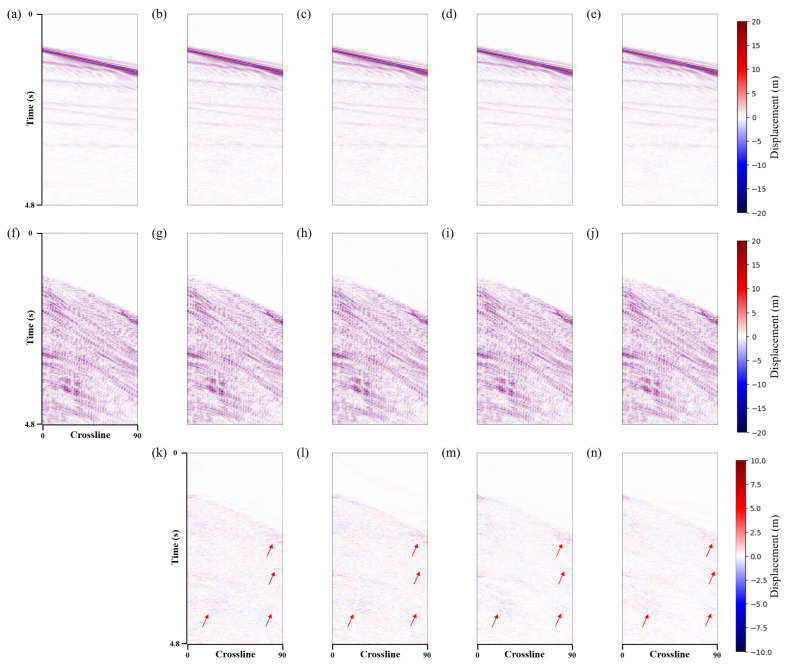
Synthetic data inline profile shear wave suppression results. (**a**) Clean data. Denoised data using the (**b**) *Unet*, (**c**) *Unet-LNCC*, (**d**) *Unet-CBAM* and (**e**) proposed method. (**f**) Field shear wave noise. Shear wave noise removed using the (**g**) *Unet*, (**h**) *Unet-LNCC*, (**i**) *Unet-CBAM* and (**j**) proposed method. (**k**–**n**) correspond to the difference between the denoised and clean data.

To better compare the denoising results, two profiles (i.e., inline and crossline) were selected for further comparison. Figure 5 and Figure 6 show the same conclusions. The proposed method can more effectively suppress the shear wave noise and has a better event continuity, with no significant signal leakage. Table 1 illustrates the corresponding S/N and SSIM values, with the proposed method also demonstrating the most favourable performance. The signal has a higher SNR and SSIM after being processed using the method presented in this paper. Based on the aforementioned analysis, the proposed method is effective in suppressing shear wave noise and achieves the separation of P-wave signals and shear wave noise in 3D synthetic data.

### 3.3. Field Data Example

**Figure 7:**
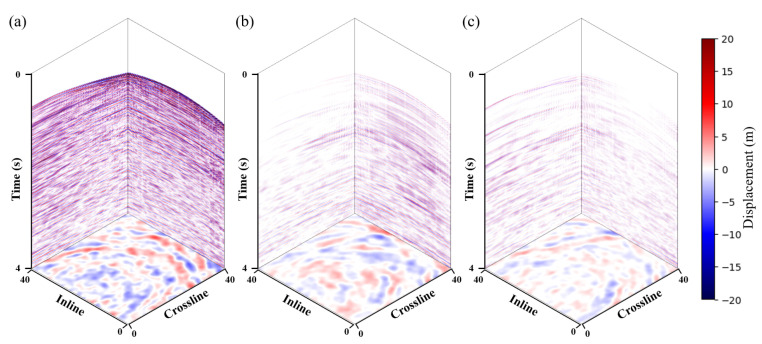
3D field data. (**a**) Vertical Z-component data. (**b**) Horizontal X-component data. (**c**) Horizontal Y-component data.

This paper further verifies the performance of the proposed method for shear wave suppression using 3D field data from an offshore area. This paper selected a portion of one receiver node’s data, for example, with a size of 2000×40×40 (Figure 7). The optimal parameters of the network, were selected through trial-and-error, where the initial learning rate was 0.001, and for every 1000 epochs, the scale of the learning rate was multiplied by 0.1 and a total of 1600 epochs were trained. The weighting parameter λ is 1 × 10^−6^.

**Figure 8:**
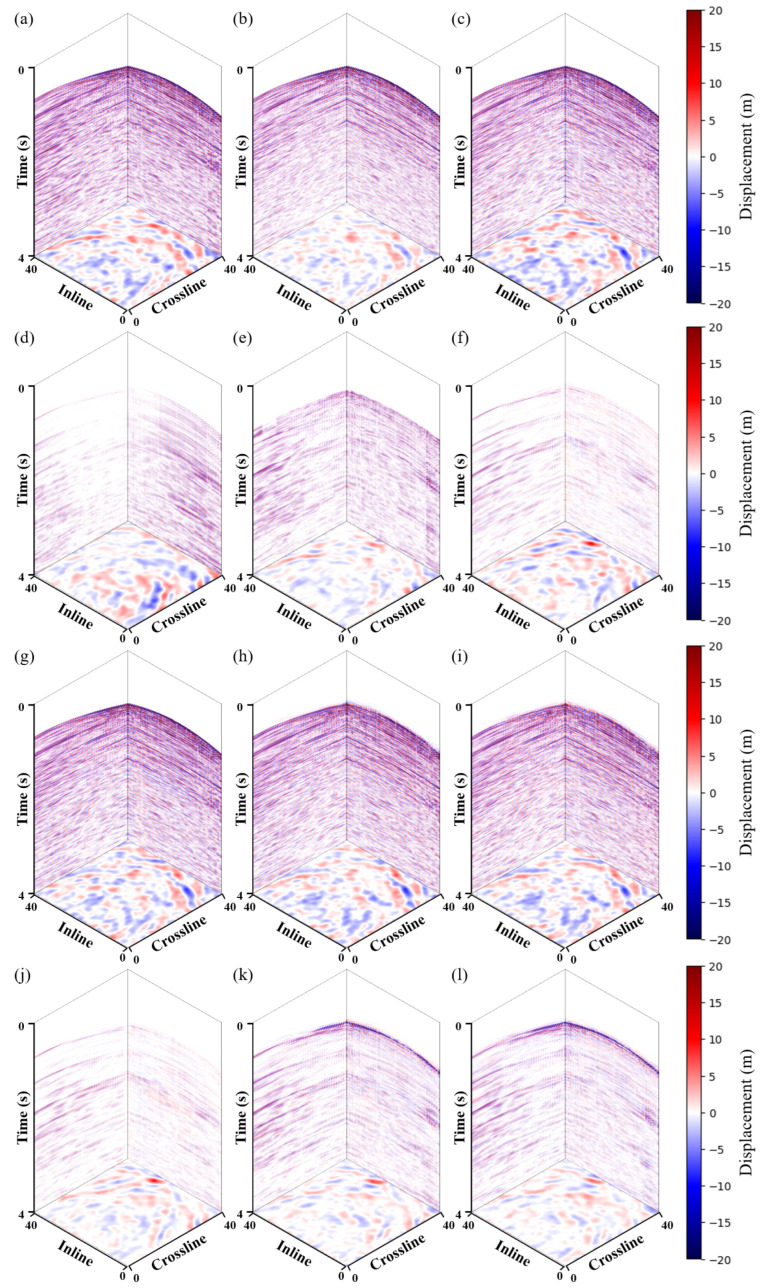
3D field data shear wave suppression results. (**a**) Noisy data. Denoised data using the (**b**) commercial software, (**c**) *Unet*, (**g**) *Unet-LNCC*, (**h**) *Unet-CBAM*, and (**i**) proposed method. (**d**) Raw horizontal XY-component. Removed shear wave noise using the (**e**) commercial software (**f**) *Unet*, (**j**) *Unet-LNCC*, (**k**) *Unet-CBAM*, and (**l**) proposed method.

**Figure 9:**
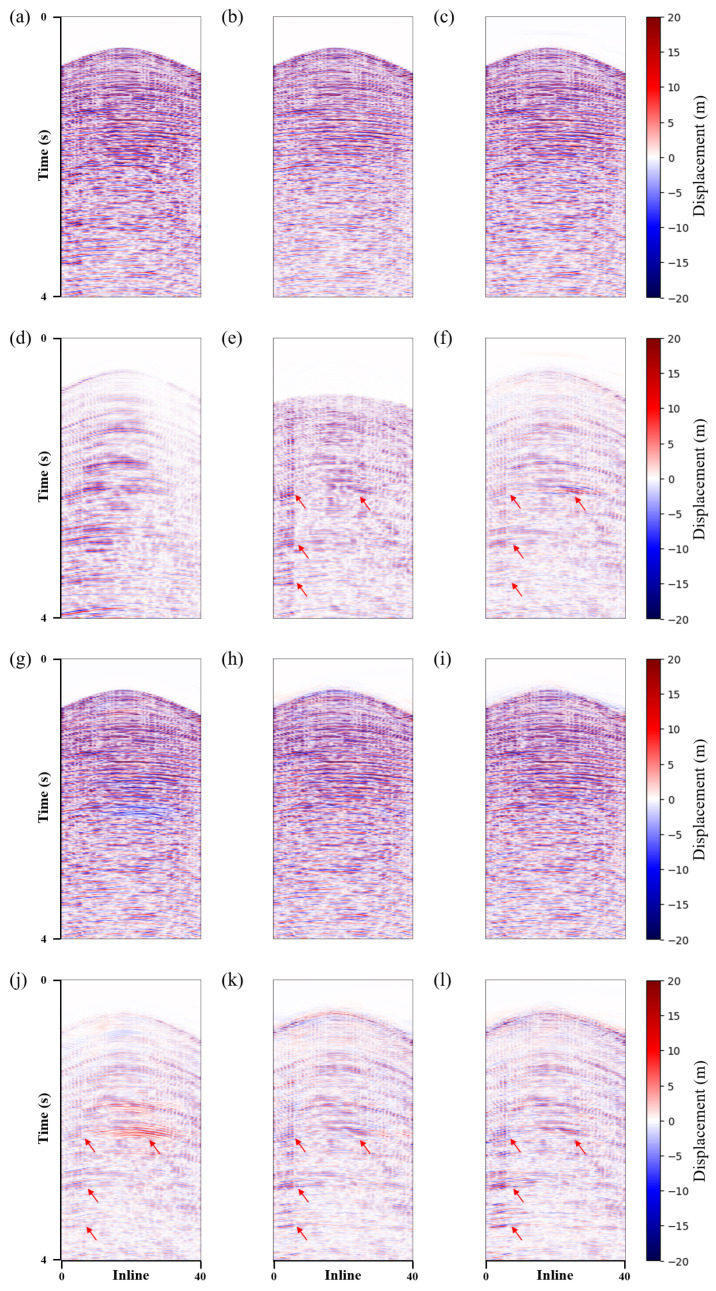
Field data crossline profile shear wave suppression results. (**a**) Noisy data. Denoised data using the (**b**) commercial software, (**c**) *Unet*, (**g**) *Unet-LNCC*, (**h**) *Unet-CBAM*, and (**i**) proposed method. (**d**) Raw horizontal XY-component. Shear wave noise removed using the (**e**) commercial software (**f**) *Unet*, (**j**) *Unet-LNCC*, (**k**) *Unet-CBAM*, and (**l**) proposed method.

**Figure 10:**
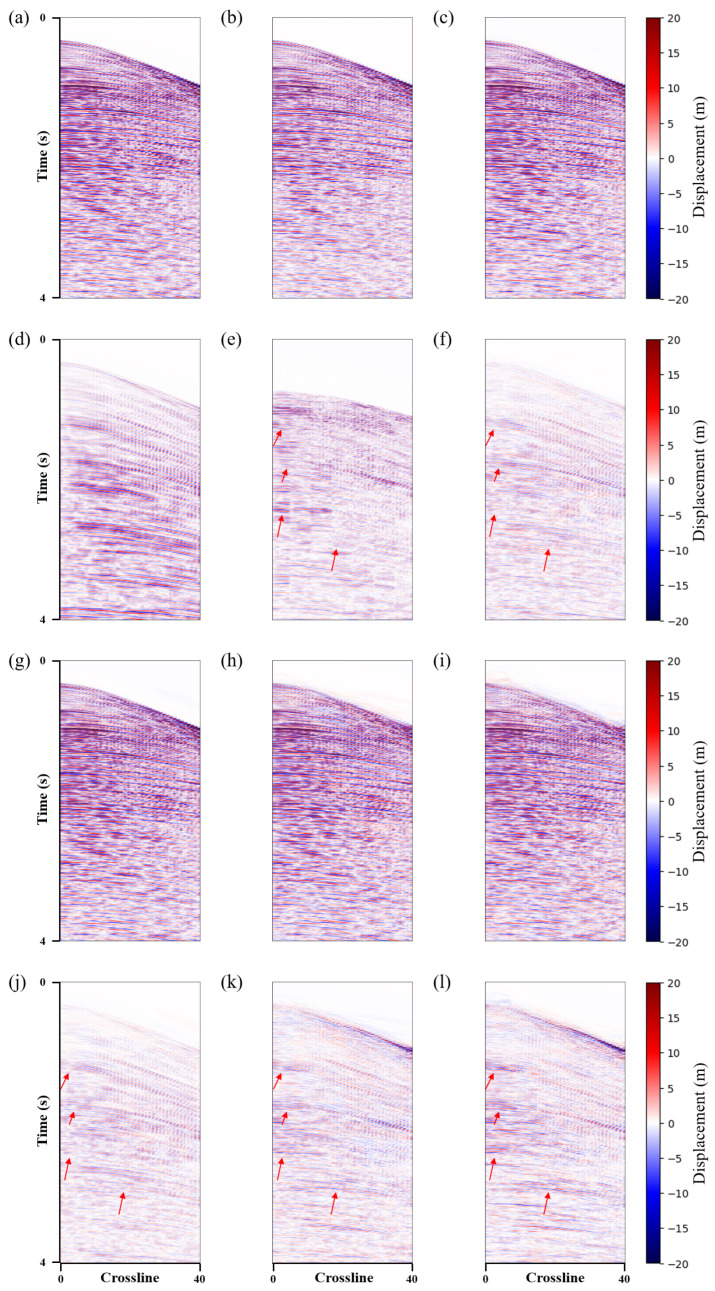
Field data inline profile shear wave suppression results. (**a**) Noisy data. Denoised data using the (**b**) commercial software, (**c**) *Unet*, (**g**) *Unet-LNCC*, (**h**) *Unet-CBAM*, and (**i**) proposed method. (**d**) Raw horizontal XY-component. Shear wave noise removed using the (**e**) commercial software (**f**) *Unet*, (**j**) *Unet-LNCC*, (**k**) *Unet-CBAM*, and (**l**) proposed method.

In the experiment on field data, this paper compared the denoising results with those of commercial software. The commercial software processes the data by first performing NMO and then suppressing the shear wave noise using adaptive matching subtraction. The method requires a P-wave velocity model, whereas our approach does not. Figure 8 shows the denoising results of the five methods and the predicted shear wave noise. Overall, among the four deep learning methods, our method better suppresses noise compared to the other three deep learning methods. The comparing deep learning methods leave residual noise in certain regions. As with the synthetic data, this paper selected two profiles in the middle in the crossline and inline directions for comparison, as shown in Figure 9 and Figure 10. As demonstrated in the Figure 9 and Figure 10, using the noise attenuation results from commercial software as a reference, it can be observed that our method can suppress noise more effectively than other deep learning methods, particularly at the areas indicated by the red arrows.

**Figure 11:**
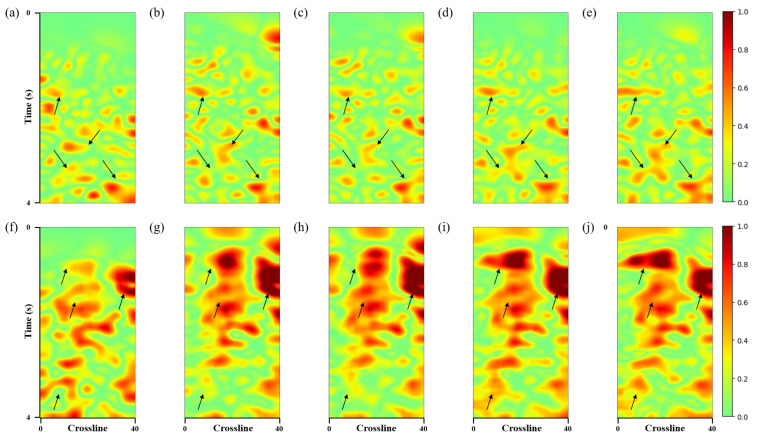
3D field data. The top row presents a map showing the local similarities between the crossline profile-predicted noise and the horizontal XY-component, and the bottom row shows a map of local similarities between the inline profile-predicted noise and the horizontal XY-component. (**a**,**f**) Data denoised using the commercial method; (**b**,**g**) data denoised using the *Unet*; (**c**,**h**) data denoised using the *Unet-LNCC*; (**d**,**i**) data denoised using the *Unet-CBAM*; (**e**,**j**) data denoised using the proposed method.

Since the shear wave noise leaks from the horizontal components, this paper calculated the local similarity between the predicted noise and the horizontal components (Figure 11). The first and second rows correspond to the computation of local similarity between the predicted noise and the corresponding horizontal components in Figure 9 and Figure 10, respectively. It can be observed that our method yielded higher local similarity values, which further demonstrates the effectiveness of our approach.

## 4. Discussion

Field OBN data contain shear wave noise that leaks from two horizontal components, therefore, efficiently processing two horizontal components is a challenge. Typically, two networks are selected to suppress the noise from the two horizontal components in two stages. Undoubtedly, training two networks is time-consuming in practical applications, and even more inefficient when dealing with 3D data. To address this problem, this paper explored how to suppress noise using both horizontal components simultaneously. One might choose to train two networks simultaneously or input the two noise models through separate channels into the network at the same time. However, this approach inevitably increases training time, and the dual-channel method does not effectively extract the features of the two noise models. Therefore, this paper proposes a dual-encoder method to resolve these problems. This way not only improves efficiency by training a single network but also allows the dual-encoder structure to simultaneously extract features from both horizontal components. During the selection of the network architecture, to strike a balance between efficiency and precision, we reduced the four downsamples of the traditional U-Net to three downsample stages.

**Figure 12:**
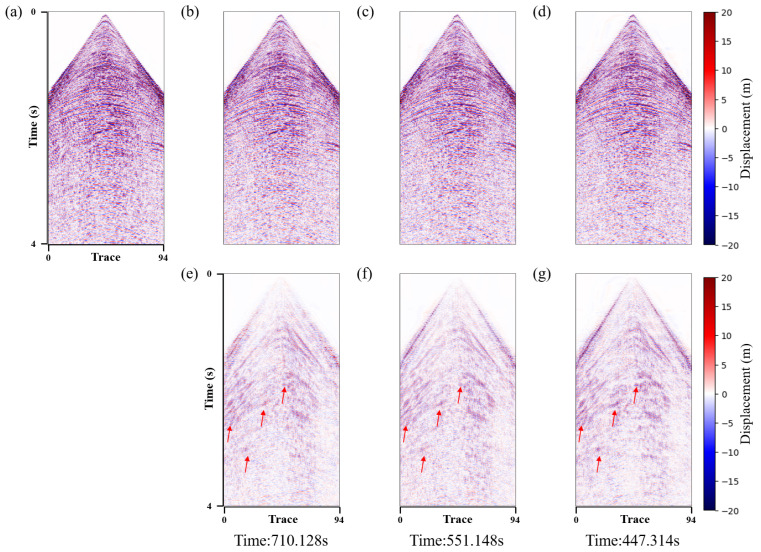
Two-dimensional field data shear wave suppression results. (**a**) Noisy data. Data denoised using the (**b**) dual network, (**c**) dual channel, and (**d**) dual encoder. Shear wave noise removed using the (**e**) dual network, (**f**) dual channel, and (**g**) dual encoder. The computation times required for the dual-network, dual-channel and dual-encoder methods were 710.128 s, 551.148 s, and 447.314 s, respectively.

This paper validated the effectiveness and efficiency of our method using 2D field OBN data. Figure 12 compares the denoised results and computation time of three approaches, including dual-network, dual-channel, and the proposed dual-encoder structures. Figure 12e–g show the noise estimations derived from the three approaches. It is evident that, in the areas indicated by the red arrow, our method demonstrates a superior ability to predict shear wave leakage. Moreover, in comparison with the other two methods, our approach is more efficient.

**Figure 13:**
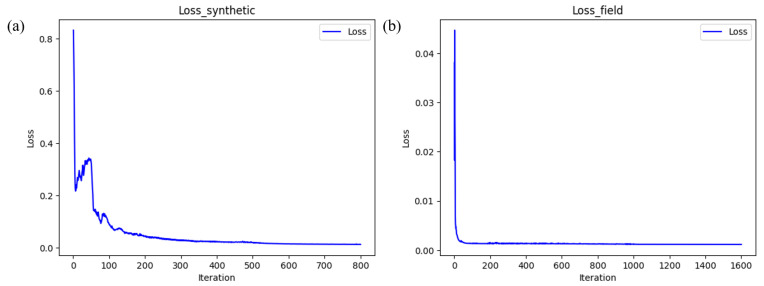
Loss function curves in network training. (**a**) Synthetic data (λ = 1e−3). (**b**) Field data (λ = 1 × 10^−6^).

Some hyperparameters in our method were chosen based on experience and experimentation. The weight of regularization in the loss function is a key hyperparameter, playing a crucial role in balancing noise suppression and signal leakage. If too large, it can easily lead to network instability, causing the predicted noise to be overly smooth. Therefore, based on our experiments, this method set λ to 1 × 10^−3^ for the synthetic data and 1 × 10^−6^ for the field data. The loss function curves are shown in Figure 13. In addition, self-supervised methods often face the issue of early stopping. In our approach, after the network was trained for a certain number of epochs and the LNCC value begins to stabilize, the current results were selected as the final outputs.

Our network has some limitations in that the network processing provides smoother results compared to the results obtained with commercial software. Commercial software can also deal with the random noise in the signal at the same time. Therefore, a future research direction is how to achieve the suppression of both types of noise at the same time using this framework. Furthermore, when the input horizontal component data are incomplete, this will also affect the noise suppression effect of the proposed method.

## 5. Conclusions

This paper developed a self-supervised denoising method for shear wave suppression in 3D OBN seismic data. It used the two horizontal components as input to the network and obtained the corresponding noise through non-linear mapping. We propose a dual-encoder U-shaped network as the fundamental framework. The CBAM was integrated into the skip connection to enhance the extraction of low-dimensional features. In addition, the introduction of the LNCC regularization term ensures the stability of the network and allows for the better separation of noise and signal. Experiments with 3D synthetic and field data demonstrate the effectiveness of the proposed method, which is capable of suppressing the shear wave noise in the vertical component. Additionally, compared to other network frameworks that simultaneously address the issue of noise in horizontal components, the proposed method is more efficient.

## Figures and Tables

**Table 1:** Quantitative comparison of denoising results from different methods on synthetic data. The **best** and second-best values are highlighted. (S/N ↑, and SSIM ↑).

	Metric	Observed	*Unet*	*Unet-LNCC*	*Unet-CBAM*	*Proposed Method*
3D data	S/N (dB) SSIM	−1.14 0.578	15.73 0.968	15.75 0.966	16.97 0.977	**17.41** **0.980**
Crossline	S/N (dB) SSIM	−0.71 0.426	16.27 0.894	16.34 0.887	18.14 **0.935**	**18.16** 0.932
Inline	S/N (dB) SSIM	−4.13 0.371	13.34 0.834	13.49 0.821	14.85 0.886	**15.18** **0.887**

## Data Availability

Data are available upon request due to restrictions, e.g., privacy or ethical restrictions.

## Abbreviations

The following abbreviations are used in this manuscript:

OBN	Ocean Bottom Node
OBC	Ocean Bottom Cable
CRGs	Common Receiver Gathers
NMO	Normal-moveout Correction
SVD	Singular Value Decomposition
DNNs	Deep Neural Networks
CBAM	Convolutional Block Attention Module
CAM	Channel Attention Module
SAM	Spatial Attention Module
MLP	Multilayer Perceptron
LNCC	Local Normalized Cross-correlation
SSIM	Structural Similarity
LS	Local Similarity
